# Functional Subunit Reconstruction of Giant Facial Congenital Melanocytic Nevi in Children With the Use of Matriderm and Skin Graft: Surgical Experience and Literature Review

**Published:** 2018-10-05

**Authors:** Nehal Mahabbat, Nawaf Alohaideb, Faris Aldaghri, Feras Alshomer, Mohamed Amir Murad

**Affiliations:** Plastic and Reconstructive Surgery Section, Department of Surgery, King Faisal Specialist Hospital and Research Centre, Riyadh, Saudi Arabia

**Keywords:** nevus, dermal, matrix, graft, face

## Abstract

**Introduction:** Facial giant congenital melanocytic nevus represents a major cosmetic deformity for the child and parents and is a challenge for the plastic surgeons to achieve best cosmetic results. Herein, we present a case of single-stage surgical reconstruction using partial-thickness scalp skin graft aided with Matriderm dermal substitute for a facial giant congenital melanocytic nevus. **Methods:** An 8-year-old boy presented with a facial giant congenital melanocytic nevus without leptomeningeal involvement. A single-stage complete excision of the nevus was performed. A split-thickness skin graft, 12/1000-inch thick, was then harvested from the anterior scalp region for reconstruction. A 1-mm Matriderm dermal substitute was first applied, on which functional subunit skin graft was then secured to cover the defect. Eyelid reconstruction was reconstructed separately using full-thickness postauricular skin grafts. **Results:** Histopathology of the excised specimen confirmed the diagnosis of congenital melanocytic nevus, with no evidence of melanoma. The donor area healed with a favorable scar and no donor site morbidity or complications such as alopecia or hypertrophic scar. The postoperative result was satisfactory with minimal residual nevus around the eye, and the patient was fully satisfied with the cosmetic and functional results. **Discussion and Conclusions:** Resection of facial congenital melanocytic nevi, followed by single-stage reconstruction using Matriderm and skin graft from the scalp, is an excellent and fast reconstructive method with promising aesthetic outcomes and greater improvement in physiological outcome, especially in the pediatric population.

Giant congenital melanocytic nevus (GCMN) is defined as a congenital melanocytic lesion involving more than 2% body surface area in infants and toddlers or a diameter of more than 20 cm in adults.^1^^,^^2^ Approximately 1 in 20,000 people are born with a large congenital melanocytic nevus (CMN) and 1 in 500,000 are born with a very large (giant) CMN,^3^^-^5 with a higher potential risk of malignant transformation during the first 20 years of life.^6^ Giant nevi on the scalp and neck might be associated with neurological disorders such as focal neurological abnormalities, neurofibromatosis, or epilepsy, known as leptomeningeal melanocytosis. Neuroimaging studies are recommended for such patients to detect associated disorders that could affect treatment and prognosis.^7^ A lesion on the face represents a major cosmetic deformity for the child and parents and is a challenge for the plastic surgeons to achieve best cosmetic results. Moreover, not all giant congenital nevi are pigmented, resulting in confusion about the management approach and follow-up. In total, 70% of melanomas are diagnosed by the age of 10 years.^7^^,^^8^ The relative risk of developing melanoma within a GCMN varies among types from 5% to 10% over one's lifetime.^9^ Hence, early prophylactic excision and reconstruction are advisable.^10^^,^^11^

The goal of treatment is complete excision with satisfactory cosmetic reconstruction. Therefore, during treatment decision, factors such as psychological effect and the risk of surgery and malignant transformation should be considered.^12^

We report here a case of a patient who underwent single-stage lesion resection and functional subunit reconstruction with the use of Matriderm acellular dermal matrix and partial-thickness skin graft harvested from the scalp, with acceptable functional and aesthetic results.

## METHODS

### Case presentation

**Figure 1:**
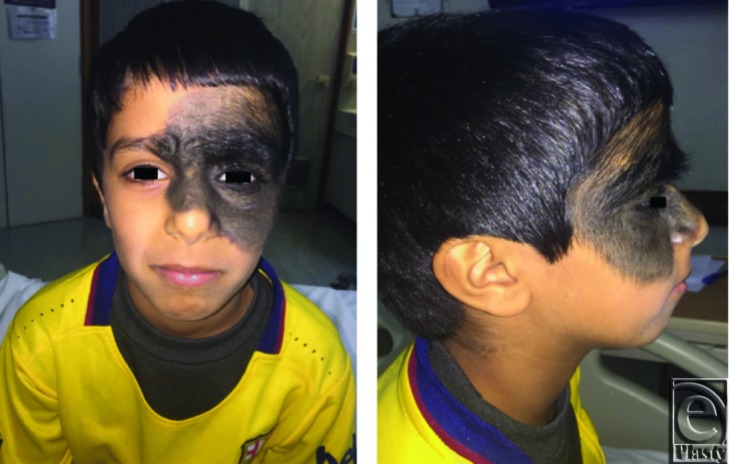
The preoperative view of the giant congenital melanocytic nevus. Extensive black lesion on the left side of the face. Note the involvement of both upper and lower eyelids on the involved side.

An 8-year-old boy presented with an extensive, large, black, hairy skin patch over the left periorbital area, forehead, cheek, and nose since birth. There was no family history of similar lesions or skin cancer. The patient had no neurological symptoms and was not taking any medications. Examination revealed a large pigmented patch, measuring approximately 13 cm in its greatest dimension on the left periorbital area and extended to cover nearly half of the face (Fig 1). There was no increase in the size or change in color of the lesion since birth, and there was no pain, itching, or discharge. No other satellite lesions were present over the body, and there were no associated congenital anomalies. Parents’ counseling indicated that the lesion was affecting his school and social activities.

### Treatment and outcomes

We performed a single-stage complete excision of the lesion under general anesthesia after discussing the surgical risks and benefits, and the potential for malignancy, with the family of the patient. Functional reconstruction was performed first with a thin Dermal Regeneration Template (Matriderm 1 mm). Next, 12/1000-inch split-thickness skin graft harvested from the anterior scalp was secured on top of it using absorbable sutures. Separate sheets of skin graft were applied to different anatomic areas following the subunit principle of reconstruction where feasible.

**Figure 2:**
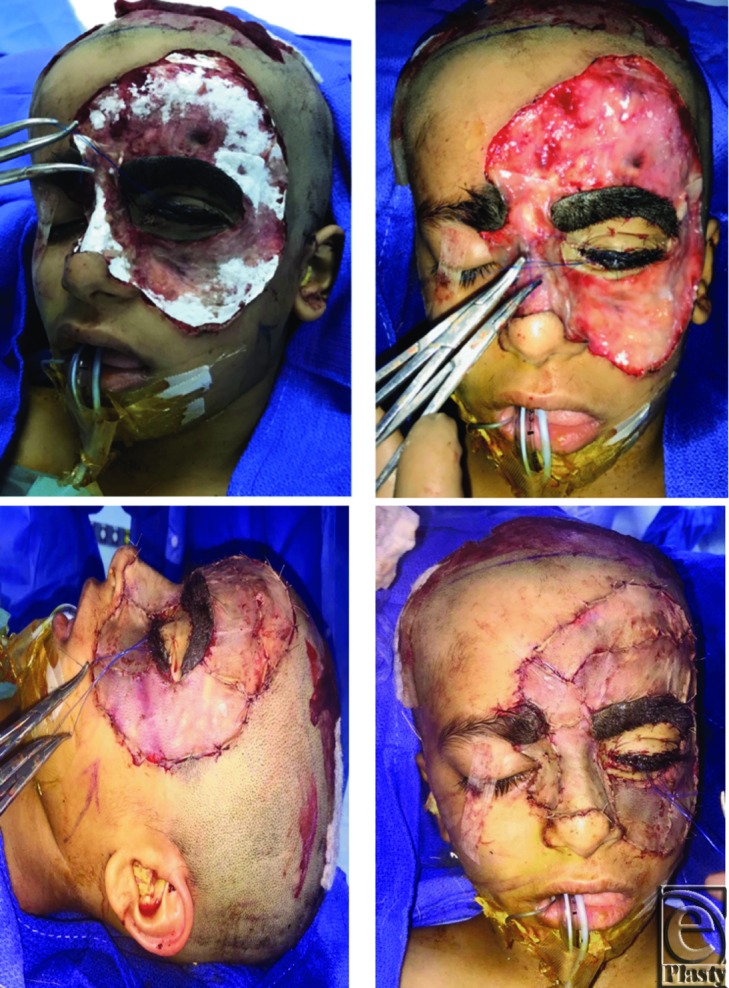
The intraoperative view of the facial lesion after excision, followed by the application of Matriderm to the excised area. Nevus involving the eyelids was reconstructed using full-thickness skin graft.

In addition, full-thickness skin grafts harvested from the postauricular region were used to cover the upper and lower eyelids (Fig 2). The grafts were then secured with tie-over bolster dressing. The eyebrow was countered with a residual nevus for subsequent reconstructive session. Dressing was changed after 5 days, which showed minimal graft loss. Further follow-ups showed the healed donor site with no donor site morbidity or complications such as alopecia or hypertrophic scar.

**Figure 3:**
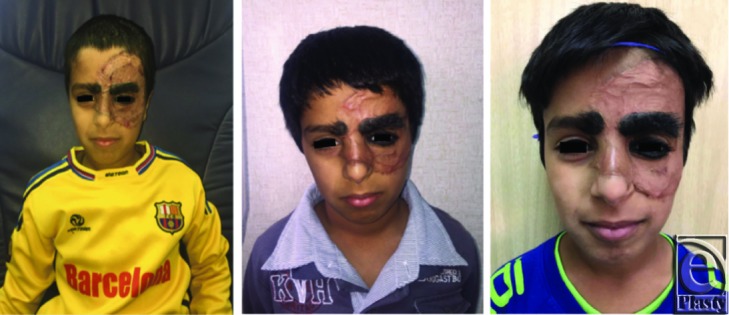
Results after a follow-up period of 6 months: (a) 2 months; (b) 4 months; and (c) 6 months. Note the maturation of the healing process of split-thickness skin graft harvested from the scalp over Matriderm. Good color match is seen together with no associated alopecia at the scalp donor site.

The postoperative result was satisfactory with excellent contour, color match, texture, and thickness to cover the giant defect created after excision. Further follow-up visits revealed that the patient and his family were fully satisfied with the cosmetic and functional results, with improvement in the child functional and social status (Fig 3).

## DISCUSSION

The management of GCMN remains controversial, with no specific guidelines. Treatment options vary on the basis of type, size, and location. Recent studies demonstrated that early excision of these giant lesions reduces the risk of malignant melanoma and the associated psychological distress in the child and parents.^13^ Several therapeutic procedures have been considered. Nonsurgical options include dermabrasion, laser ablation, curettage, and chemical peel. Surgical options include staged excision with primary closure, skin graft, flap, skin substitute, or tissue expansion reconstruction^14^; however; surgical excision remains the standard of care. Since it is impossible to eliminate the risk of malignant transformation, GCMN removal is a reconstructive and aesthetic procedure, rather than prophylactic surgery.^15^^-^17 Tissue expansion is the most commonly used modality for resurfacing the defect area after excision, with minimal donor site morbidity.^18^ However, infection, hematoma, expander exposure, and implant failure are the most common complications of tissue expansion,^19^ and their incidence is often reported to be higher in children.^20^^,^^21^ Skin grafting is recommended for lesions involving aesthetic areas such as the face, ear, neck, hand, and foot. Usually, a supraclavicular graft is the first choice for facial reconstruction; however, to address the issue of color match, which is a challenge with skin grafts, we used the scalp skin as a donor site.

Dermal Regeneration Template (Matriderm) is a single-use 3-dimensional matrix composed of native, structurally intact collagen fibrils and elastin for supporting dermal regeneration. The collagen is obtained from bovine dermis and contains the dermal collagen types I, III, and V. The elastin is obtained from bovine nuchal ligament by hydrolysis. It serves as a scaffold in the skin reconstitution and modulates scar tissue formation.^22^

The use of acellular dermal matrix in the treatment of facial cutaneous defects has been vastly investigated in multiple fields, especially in burn defects, with great functional results.^23^ Integra was shown to be of promising aesthetic results when it was used for various facial giant hairy nevus defect reconstruction. However, staged reconstruction at certain times might delay the finalized outcome.^24^ We used Matriderm as a Dermal Regeneration Template in the defect reconstruction, avoiding the need for multiple stages or the complications associated with other options such as tissue expanders or the complexity of different reconstructive processes as microsurgical free tissue transfer with promising functional and aesthetic outcomes.

## CONCLUSION

Surgical excision of facial CMNs, followed by single-stage reconstruction using Matriderm and subunit skin graft from the scalp, is an effective modality with promising aesthetic results at a shorter time and faster return to daily activities together with improved psychological function, especially in the pediatric population.
